# The effect of ‘paying for performance’ on the management of type 2 diabetes mellitus: a cross-sectional observational study

**DOI:** 10.3399/bjgpopen20X101021

**Published:** 2020-04-01

**Authors:** Raymond O'Connor, Rory O'Driscoll, Jane O'Doherty, Ailish Hannigan, Aoife O'Neill, Conor Teljeur, Andrew O'Regan

**Affiliations:** 1 Senior Research Fellow, Department of General Practice, Graduate Entry Medical School, University of Limerick, Plassey, Limerick, Republic of Ireland; 2 GP, Kenmare Primary Health Centre, Kenmare, Kerry, Republic of Ireland; 3 Research Assistant, Department of General Practice, Graduate Entry Medical School, University of Limerick, Plassey, Limerick, Republic of Ireland; 4 Professor of Medical Statistics, Graduate Entry Medical School, University of Limerick, Plassey, Limerick, Republic of Ireland; 5 PhD Candidate, Department of Mathematics and Statistics, University of Limerick, Plassey, Limerick, Republic of Ireland; 6 Chief Scientist, Health Information and Quality Authority, Dublin, Republic of Ireland; 7 Senior Lecturer, Department of General Practice, Graduate Entry Medical School, University of Limerick, Plassey, Limerick, Republic of Ireland

**Keywords:** pay for performance, diabetes mellitus, quality improvement, reimbursement, incentive, general practice, primary healthcare

## Abstract

**Background:**

The ‘cycle of care’ (COC) pay for performance (PFP) programme, introduced in 2015, has resourced Irish GPs to provide structured care to PCRS eligible patients with type 2 diabetes mellitus (T2DM).

**Aim:**

To investigate the effect of COC on management processes.

**Design &setting:**

Cross-sectional observational study undertaken with two points of comparison (2014 and 2017) in participating practices (Republic of Ireland general practices), with comparator data from the United Kingdom National Diabetes Audit (UKNDA) 2015–2016.

**Method:**

Invitations to participate were sent to practices using a discussion forum for Health One clinical software. Participating practices provided data on the processes of care in the management of patients with T2DM. Data on PCRS eligible patients was extracted from the electronic medical record system of participating practices using secure customised software. Descriptive analysis, using IBM SPSS Statistics for Windows (version 25), was performed.

**Results:**

Of 250 practices invited, 41 practices participated (16.4%), yielding data from 3146 patients. There were substantial improvements in the rates of recording of glycosylated haemoglobin ([HbA1c] 53.1%–98.3%), total cholesterol ([TC] 59.2%–98.8%), urinary albumin:creatinine ratio ([ACR] 9.9%–42.3%), blood pressure ([BP] 61.4%–98.2%), and body-mass index ([BMI] 39.8%–97.4%) from 2014 to 2017. For the first time, rates of retinopathy screening (76.3%), foot review (64.9%), and influenza immunisation (69.9%) were recorded. Comparison of 2017 data with UKNDA 2015–2016 was broadly similar.

**Conclusion:**

The COC demonstrated much improved rates of recording of clinical and biochemical parameters, and improved achievement of targets in TC and BP, but not HbA1c. Results demonstrate substantial improvements in the processes and quality of care in the management of patients with T2DM.

## How this fits in

Incentivising general practice teams by PFP has been suggested as an option to help manage PCRS eligible patients with T2DM. The introduction of the COC programme in the Republic of Ireland for PCRS eligible patients presents the opportunity to investigate this. In the present study, substantial improvement was shown for rates of recording of important clinical and biochemical parameters and in the achievement of some but not all targets, supporting the introduction of PFP. Such a policy can be readily introduced into other countries with a functioning primary care system but lacking chronic disease management programmes.

## Introduction

The detrimental health effects of T2DM complications on the patient, coupled with alarming global expenditure on the disease’s management, have made the question of how best to manage T2DM a central clinical and health planning topic.^1^ Intensive control of risk factors, such as glucose levels, lipids, and elevated BP lowers the incidence of complications in diabetes.^2,3^ Incentivising clinicians by PFP has been suggested as a strategy to achieve this.^4^ The World Health Organization advocates that management of patients with T2DM should be community-based in primary healthcare settings, with an established referral and back-referral system.^5^ An example of such an approach is the Irish Health Service Executive Midland Diabetes Structured Care Programme.^6^ This has provided high-quality, primary care-led management of patients with T2DM since its inception in 1998.^6^ In the UK, community-based services for managing patients with T2DM have been extensively evaluated and there is evidence to support their effectiveness.^7^

There has been much debate in the recent literature about the advantages and disadvantages of PFP. Its overarching purpose is to align payment incentives with health system objectives related to quality, care coordination, health improvement, and efficiency by rewarding achievement of targeted performance measures.^8^ Systematic reviews have found that PFP programmes result in the full spectrum of possible effects for specific targets, from absent or negligible to strongly beneficial.^9,10^ Another systematic review reported modest benefits and advised caution with the implementation of such schemes.^11^ In the UK, a PFP scheme that covers the entire population called the Quality and Outcomes Framework (QOF) has been in existence since 2004. It is one of the largest in the world.^12^

In the Republic of Ireland, approximately 37% of the population are eligible for free GP care,^13^ and the payments for this are made to GPs by the Primary Care Reimbursement Service (PCRS). From a payment perspective, such patients are referred to as ’PCRS eligible‘. The introduction of a diabetes PFP, the COC, in the Republic of Ireland in October 2015 for PCRS eligible patients presented the opportunity to investigate its effects on the management of the condition in general practice. GPs are paid a one-off registration fee of 30 EUR followed by 100 EUR per year for two patient visits. Assuming that each visit would cost approximately 20 EUR in staff costs and other practice overheads, there is some financial incentive for practices to carry out this work.

The overarching aim of this study was to investigate the effect of COC on processes and outcomes of care, and to compare with the UKNDA 2015 –2016.

## Method

### Method, setting and study design

In the Republic of Ireland, it is estimated that there are approximately approximately 207 000 people with T2DM around the time of the study,^14–16^ and 103 800 of these (50.1%) were enrolled in the COC in 2018.^17^ The remainder are PCRS ineligible patients and make their own care arrangements, which they pay for. They are not covered in this study. All patients whose records were examined in this study were coded for T2DM using a unique Health One code, which is linked to the International Classification of Primary Care (ICPC) and the 10^th^ edition of the International Classification of Diseases (ICD-10) coding systems. The authors compared results with the UKNDA 2015–2016;^18^ This is an audit of the QOF funded PFP scheme in the UK and serves as a useful comparator. The known prevalence of T2DM in the UK was approximately 3.3% at the time of the UKNDA 2015–2016.^19^ The QOF system and payments are further explained in the supplementary data.

This was a multipractice retrospective study of patient data comparing two time points, before (2014) and after (2017) the introduction of COC. There were four specific objectives: 1) to investigate if there was any improvement in the process of care reflected in the rates of recording of essential clinical and biochemical parameters, namely BP, HbA1c, ACR, serum creatinine (Cr), TC, BMI (weight [kg] divided by height [m^2^]), foot review, retinopathy screening, and influenza immunisation status. 2) To compare outcomes of care in the form of treatment target achievement for HbA1c, TC and BP for patients in 2014 and 2017 where such data existed for both years. 3) To provide a baseline for ongoing monitoring of the COC, as well as identifying any other data that could be captured prospectively. 4) To compare findings with data from the UKNDA 2015–2016^18^ from the same time period in terms of completeness of data recorded and treatment target achievement for HbA1c, TC, and BP in 2017.

### ​Participants and recruitment

In 2015 there were 2932 GPs and 1700 practice nurses working in 1734 practices in the Republic of Ireland, approximately 94% of which are computerised.^20^ It is estimated that Health One clinical software is used by approximately 400 of these practices.^21^ Approximately 250 of these use a discussion forum for the software. The percentage of these practices that are registered to provide COC services to their patients is not known. In 2019 in the Republic of Ireland, approximately 2215 GP practices were registered with the Irish PCRS to provide COC services to their patients.^22^

A letter of invitation to participate in the study was circulated to all practices using the discussion forum in mid-March 2018. Follow-up letters were issued 1 and 2 months later.

### ​Data extraction

The study instrument was designed by 2 GPs on the research team to extract the relevant information at the two time points from practices using Health One clinical software. It was initially piloted in six GP practices and was subsequently adapted and refined, based on feedback from the participants.

Three types of data were collected:

1. Demographic data on participating GPs and their practices. This included the age of the senior GP lead in the practice, the sex of all participating GPs, and the location of the practice, from which the area deprivation score was calculated. This allowed comparison of data with a nationally representative sample (Supplementary Table 2);
2. Demographic data on patients with T2DM. This included the age and sex of patients, and allowed the authors to compare results with those of the UKNDA 2015– 2016^18^ (Supplementary Table 3).
3. Clinical data. No end point outcomes (such as death or myocardial infarction) were collected. Processes of care included the rates of recording and the actual levels of the clinical and biochemical parameters listed in the setting and study design section.

### ​Data analysis

Data from each practice were anonymised and amalgamated into a single master file. Where multiple values were available for some clinical parameters over the 12 -month study period, a mean value was used to summarise the parameter for an individual patient. Descriptive statistics were used to characterise participating practices, patient demographics, and clinical data. Descriptive statistics were also used to compare the care processes in COC with those from the UKNDA 2015–2016 .^18^ Clinical data was compared over time for patients who had data in both 2014 and 2017. Due to the low numbers of patients with data recorded in 2014 for some clinical parameters, comparisons were limited to HbA1c, TC, and BP. McNemar’s test for paired nominal data was used to test for differences in the percentage achieving targets over time (2014 and 2017) for HbA1c, TC, and BP. The UKNDA 2015–2016^18^ targets of HbA1c ≤58 mmol/mol, TC <5 mmol/litre, and BP≤140/80 mmHg^18,23,24^ were used for data from 2014 and 2017. These targets are broadly in line with Irish target values at the time of the study.^6^ IBM SPSS Statistics for Windows (version 25) was used to carry out the analysis and a 5% level of significance used for all tests.

## Results

**Figure 1.**
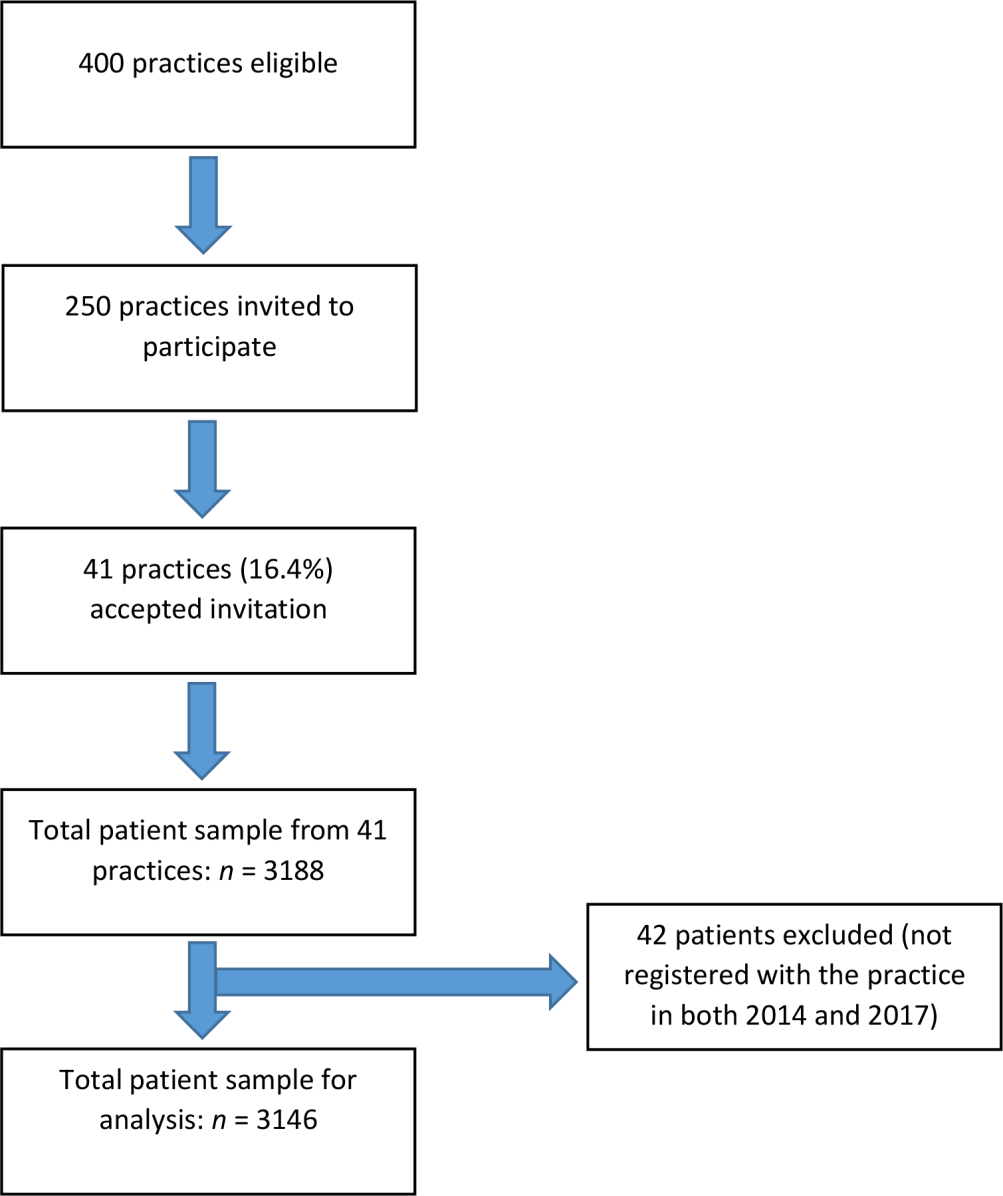
PRISMA diagram of participant inclusion.

Forty-one practices agreed to participate in the study, giving a response rate of 16.4% from practices on the Health One discussion forum, and yielding data on 3146 patients with T2DM who were registered with the practices in both 2014 and 2017 (Figure 1). The characteristics of the sample of GPs studied were similar to a nationally representative sample^20^ (Supplementary Table 2). The age and sex profile of the patients was similar to the UKNDA 2015–2016^18^ (Supplementary Table 3), and also to an Irish population of patients with T2DM^6^ (Supplementary Table 4).

### ​Processes of care

Table 1 shows the rates of recording of clinical and biochemical parameters in 2014 and 2017, and compares them to the UKNDA 2015–2016.^18^ The proportion of parameters recorded improved substantially from a low level or from not being recorded, to a level where the figure was now comparable with the UKNDA 2015–2016^18^ figures.

**Table 1.** Comparison of care processes over time (2014–2017) to UK National Diabetes Audit 2015–2016

**Care processes**	**Pre-COC** (2014), *n*= 3146, *n* (%)	**COC (2017),***n*= 3146, *n* (%)	**UK National Diabetes Audit (2015–2016**), *n*= 2 500 698, *n* (%)
HbA1c	53	98	95
Total cholesterol	59	99	93
Creatinine	9	80	9 5
ACR	10	42	67
BP	61	98	9 6
BMI	40	97	82
Attending or referred to retinopathy screening	NR	76	NR
Foot review	NR	6 5	87
Influenza immunisation offered	NR	70	NR

ACR = urinary albumin:creatinine ratio. BMI = body mass index. BP = blood pressure. COC = cycle of care. HbA1c = glycosylated haemoglobin. NR = not recorded.

### ​Outcomes of care

**Figure 2.**
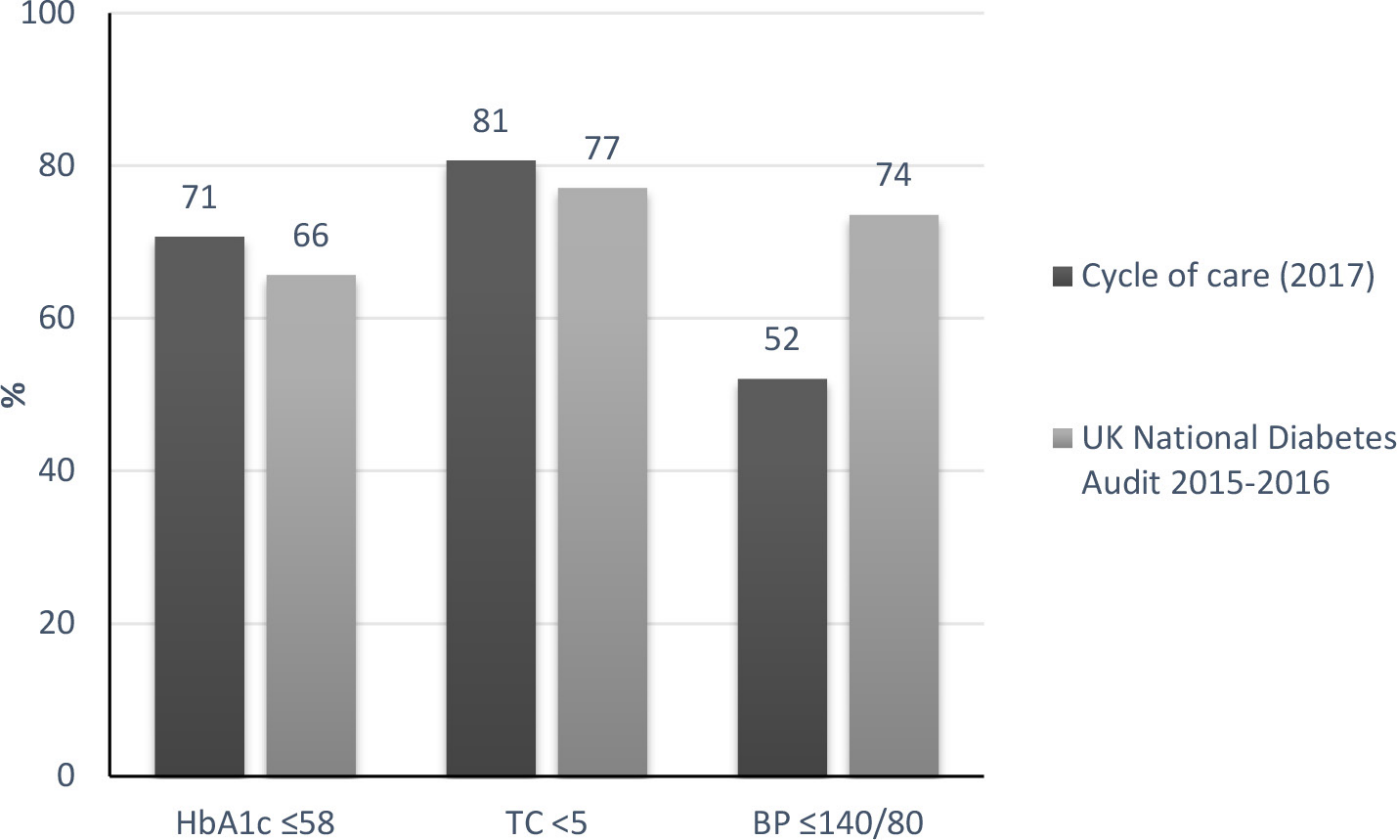
Comparison of percentage of patients achieving treatment targets in 2017 cycle-of-care sample compared with the UK National Diabetes Audit (2015–2016). BP = blood pressure. HbA1c = glycosylated haemoglobin. TC = total cholesterol.

The comparison of the percentage of patients in 2017 achieving treatment targets for HbA1c, BP, and TC in the COC sample compared with the UKNDA 2015–2016^18^ is shown in Figure 2.

COC was better for achieving target values in HbA1C and TC (71% versus 66% and 81% versus 77%, respectively), but not with BP (52% versus 74%).

Table 2 shows a comparison of the achievement of treatment target values of HbA1c, TC, and BP for the subset of study patients who had data recorded in both 2014 and 2017. The changes over time in the percentages achieving treatment targets were statistically significant for all three parameters (*P*<0.001), with the percentage of patients achieving targets improving over time for TC and BP, but decreasing slightly for HbA1c.

**Table 2.** Comparison of values of HbA1c, TC, and BP for patients with data recorded for 2014 and 2017

**Parameter and target range**	**Total sample size, *n*^a^**	**2014, ** **% within target range**	**2017, ** **% within target range**	***P* value ^b^**
HbA1c ≤58 mmol/mol	1665	76	72	< 0.001
TC <5 mmol/litre	1851	76	83	<0.001
BP ≤140/80 mmHg	1904	42	55	<0.001

^a^Patients with data measured in 2014 and 2017. ^b^From McNemar’s test for paired nominal data.

## Discussion

### ​Summary

To the authors’ knowledge, this is the first study to examine the effect of the COC initiative on the standard of care of patients with T2DM in Irish general practice. Results demonstrate a substantial improvement in the processes of care since the introduction of the programme, such that it is now directly comparable with the UK system, which has been much longer established. As well as improving the rates of recording of important clinical and biochemical parameters, other equally important parameters (such as foot care, influenza vaccination, and retinopathy screening) started to be measured, or those measurements began to be recorded in a manner that allowed subsequent population analysis. Such parameters may have been recorded in the past as free text in the patient’s file.

Demonstrated improvements in recording essential data as part of care for this sample of patients eligible under the PCRS with T2DM provided by general practice teams is an important improvement in care provided, as is improvement in targets achieved, particularly in TC and BP, but not in HbA1c, levels.

These data raise concerns within the health system and for Irish society in relation to the majority of people with T2DM, who are actually not eligible or enrolled under the COC. Data also require reflection and response to the observed failure to further reduce HbA1c levels within the COC.

### ​Strengths and limitations

Strengths of the present study include the use of appropriate methodology, delivering clear results in a very important area of care. The authors used an automated data collection process where the data was gathered directly from practice computer systems. The characteristics of the sample of GPs studied were similar to those of a nationally representative sample, making the data generalisable. All patients involved in the programme were analysed. The demographic characteristics of the study patient population was very similar to that of an Irish and UK population with T2DM, with which comparison was made. Results provide a baseline for ongoing monitoring of the COC.

Limitations were that the authors only have data from 2 years, and that the low rate of recording in 2014 limits comparisons of all outcomes over time. Other limitations were that PCRS-ineligible patients in the practices who were not covered by the COC were not studied, and at the time of the study only 45.5% of patients with T2DM were covered by the COC. This was an observational study with no control group of practices that did not become involved in the COC programme. The study outcomes were limited to the processes of care and measurements, and did not include long-term morbidity and mortality indicators. Another limitation was that the authors only invited practices that used Health One clinical software to participate using a discussion forum. The response rate of 41 practices was only 16.4% of those eligible to participate.

### ​Comparison with existing literature

The results of the present study are more favourable to a similar before-and-after study ofPFP for chronic illness conducted among small practices in New York.^25^ Elsewhere, it has been suggested that the impact of PFP is often difficult to assess because of patient factors, as well as practice organisational factors.^26^ The involvement of target users and stakeholders (such as GPs) at the design stage of developing the PFP process may enhance their commitment to the programme with good effect.^9,27^ After 1 year, for example, Kirschner *et al* found significant improvement for the process indicators for all chronic conditions affected by their PFP.^28^ While one study concluded that it would be more cost effective to discontinue PFP in primary care and *’return all incentive payments to the NHS’*,^29^ removal of financial incentives for certain elements of performance indicators covered by the QOF was associated with an immediate decline in performance on quality measures.^30^ In the most recent report on the QOF system, NHS England concluded that it had a modest effect on health outcomes and should be continued, but in a way that enables more holistic, person-centred care.^31^

Comparison of the data from the subset of patients who had clinical data collected in both 2014 and 2017 shows that the percentage of those achieving treatment target values for BP and TC increased over time, but decreased slightly for HbA1c. Therefore, the overall effect on the standards of care of these important risk factors was positive. Systematic review of PFP programmes in the UK has suggested that PFPs be accompanied by long-term monitoring and evaluation,^32^ and such recommendations should apply to COC also. The reasons for the slight disimprovement in HbA1c values could be related to a number of factors. First, the percentage of patients in whom it was recorded increased from 53% in 2014 to 98% in 2017 (Table 1). One effect of this would be that a greater number of patients whose control was poorer and who were not being monitored under the old system were now being seen regularly. Furthermore, overall glucose control was good, with 76% of the population studied being in the target range of ≤58 mmol/mol in 2014 compared with 72% in 2017 (Table 2). Therefore, GPs may have focused in other areas that were easier to manage, such as cholesterol treatment (levels in the target range improved from 76% in 2014 to 83% in 2017) and BP (levels in the target range improved from 42% in 2014 to 55% in 2017) (Table 2). Failure to improve glucose control might also indicate a degree of ‘therapeutic inertia’ among the treating GPs. This is defined as the ’failure to advance therapy when appropriate’ and has a multiplicity of causes.^33^ Focused educational resources are being developed to combat this.^33^

While there was a relatively low level of BP readings in the target range (55% in 2017), this figure had improved by 30% from 2014. It would be expected to improve further with time as practices became more adept at regularised diabetes care and, in time, become similar to that of the UK, where PFP has been in practice since 2004.

### ​Implications for research and practice

A controlled study looking at the effects on practices that did not sign up to the initiative would show more clearly whether the effect seen was due to the COC initiative. This study supports use of focused and coordinated PFP programmes, which reward a pattern of data collection such as in this case, and not just individual measures such as weight and BP. PFP supports standardised data collection and therefore the ability to analyse it, as payment is only made if data is recorded appropriately in the correct software field. Additional diabetes data that can now be compared prospectively in the Irish context include foot examination, retinopathy referral, and influenza immunisation offered to patients. Simple PFP programmes like the COC can be readily introduced in other countries with a functioning primary care system but lacking structured chronic disease management systems.

Based on others’ experience with PFP schemes, close monitoring and audit of process and outcomes, with ongoing feedback to participating practitioners (similar to the Irish Health Service Executive Midlands Diabetes Structured Care Programme),^6^ will help refine the process to ensure COC reaches its maximal potential for optimising patient care. The ready availability of complementary services, such as retinopathy screening, can only enhance the care that the COC and these services provide to the patient.

In this study, substantial improvement was shown for rates of recording of important clinical and biochemical parameters, and in achievement of some but not all targets. This supports the introduction of PFP for the management of T2DM and likely other chronic diseases in primary care. Such a policy is readily transferrable to other countries with a functioning primary care system but lacking chronic disease management programmes.
